# Loop Diuretics Inhibit Ischemia-Induced Intracellular Ca^2+^ Overload in Neurons *via* the Inhibition of Voltage-Gated Ca^2+^ and Na^+^ Channels

**DOI:** 10.3389/fphar.2021.732922

**Published:** 2021-09-15

**Authors:** Christopher Katnik, Javier Cuevas

**Affiliations:** Department of Molecular Pharmacology and Physiology, Morsani College of Medicine, University of South Florida, Tampa, FL, United States.

**Keywords:** bumetanide, ethacrynic acid, voltage-gated channels, sodium, calcium, neurons, ischemia, acidosis

## Abstract

One consequence of ischemic stroke is disruption of intracellular ionic homeostasis. Intracellular overload of both Na^+^ and Ca^2+^ has been linked to neuronal death in this pathophysiological state. The etiology of ionic imbalances resulting from stroke-induced ischemia and acidosis includes the dysregulation of multiple plasma membrane transport proteins, such as increased activity of sodium-potassium-chloride cotransporter-1 (NKCC-1). Experiments using NKCC1 antagonists, bumetanide (BMN) and ethacrynic acid (EA), were carried out to determine if inhibition of this cotransporter affects Na^+^ and Ca^2+^ overload observed following *in vitro* ischemia-acidosis. Fluorometric Ca^2+^ and Na^+^ measurements were performed using cultured cortical neurons, and measurements of whole-cell membrane currents were used to determine target(s) of BMN and EA, other than the electroneutral NKCC-1. Both BMN and EA depressed ischemia-acidosis induced [Ca^2+^]_i_ overload without appreciably reducing [Na^+^]_i_ increases. Voltage-gated Ca^2+^ channels were inhibited by both BMN and EA with half-maximal inhibitory concentration (IC_50_) values of 4 and 36 μM, respectively. Similarly, voltage-gated Na^+^ channels were blocked by BMN and EA with IC_50_ values of 13 and 30 μM, respectively. However, neither BMN nor EA affected currents mediated by acid-sensing ion channels or ionotropic glutamatergic receptors, both of which are known to produce [Ca^2+^]_i_ overload following ischemia. Data suggest that loop diuretics effectively inhibit voltage-gated Ca^2+^ and Na^+^ channels at clinically relevant concentrations, and block of these channels by these compounds likely contributes to their clinical effects. Importantly, inhibition of these channels, and not NKCC1, by loop diuretics reduces [Ca^2+^]_i_ overload in neurons during ischemia-acidosis, and thus BMN and EA could potentially be used therapeutically to lessen injury following ischemic stroke.

## Introduction

Preservation of neuronal [Ca^2+^]_i_ and [Na^+^]_i_ homeostasis is dependent on ATPases, ion exchangers and cotransporters, and disruption of these during ischemia has a major impact on cell survival. For example, the Na^+^-K^+^-Cl^-^ cotransporter 1 (NKCC1) (X. Chen et al., 2008; Luo et al., 2008) promotes neuronal [Na^+^]_i_ overload upon reoxygenation, triggering reverse-mode operation of the Na^+^/Ca^2+^ exchanger, NCX1, Ca^2+^ influx, and cell death (Pignataro et al., 2004; Shono et al., 2010). In contrast, upregulation of the endoplasmic reticulum Ca(2+)-ATPases (SERCA2b subtype) in hippocampal neurons decreases Ca^2+^ store dysfunction and is neuroprotective during oxygen-glucose deprivation (Kopach et al., 2016).

Ionic imbalances are also a contributing factor to the acidotoxicity observed following cerebral ischemia (Siesjo, 1988). Our laboratory has shown that acidosis synergistically potentiates the intracellular Ca^2+^ dysregulation evoked by ischemia in cortical neurons, enhancing neuronal death (Mari et al., 2010). The acid-sensing ion channel, ASIC1a, contributes to this potentiation, but these channels alone cannot account for the long-lived synergy (Mari et al., 2010), since ASIC1a rapidly inactivates and release of Ca^2+^ from intracellular stores was also observed following ASIC1a activation (Herrera et al., 2008; Mari, Katnik, and Cuevas, 2010). It is of significant interest to identify other contributors to this synergistic potentiation of [Ca^2+^]_i_ dysregulation during ischemia and acidosis since these may, in part, account for the expansion of the ischemic lesion following stroke.

One possible molecular mechanism for this long-lived ionic imbalance during ischemia-acidosis is the prolonged activation of NKCC1. The role of NKCC1 during stroke and ischemia has been previously studied using the NKCC1-selective inhibitor, bumetanide. Bumetanide was shown to reduce reperfusion injury following focal cerebral ischemia in rats, presumably due to the inhibition of NKCC1 (Wang, Huang, He, Ruan, and Huang, 2014). Chronic bumetanide treatment following stroke has been shown to enhance neurogenesis and behavioral recovery in rats, although this effect was not unequivocally linked to NKCC1 (Xu et al., 2016). *In vitro* experiments have shown bumetanide can inhibit ischemia-induced [Na^+^]_i_ and [Ca^2+^]_i_ dysregulation in both neurons and astrocytes (X. Chen et al., 2008; Kintner et al., 2007; Lenart, Kintner, Shull, and Sun, 2004). These effects of bumetanide were suggested to be due to block of NKCC1.

Experiments were carried out to determine if inhibition of NKCC1 with bumetanide or ethacrynic acid alters ischemia-acidosis evoked [Na^+^]_i_ and [Ca^2+^]_i_ overload in neurons. Fluorometric Na^+^ and Ca^2+^ measurements showed that bumetanide and ethacrynic acid inhibited ischemia-acidosis induced [Ca^2+^]_i_, but not [Na^+^]_i_, overload. The effects of bumetanide and ethacrynic acid were sufficiently different to suggest that these compounds were acting on distinct off-target sites. Whole-cell membrane current measurements indicated that both bumetanide and ethacrynic acid inhibit voltage-gated calcium and voltage-gated sodium channels, but that neither affects glutamatergic ionotropic receptors or acid-sensing ion channels. Ethacrynic acid was found to be a more efficacious inhibitor of VGCC than bumetanide, which may explain why it has a greater effect on ischemia-acidosis evoked [Ca^2+^]_i_ overload. The effects of bumetanide on ischemia-acidosis evoked [Ca^2+^]_i_ overload may in part explain the benefits of this compound following stroke. However, results presented here suggest that ethacrynic acid may be a superior compound for reducing stroke injury.

## Methods

### Primary Rat Cortical Neuron Preparation

Cortical neurons from embryonic (E18) rats were isolated and cultured as previously described (Katnik, Guerrero, Pennypacker, Herrera, and Cuevas, 2006). Briefly, excised brains were digested with 0.25% trypsin. Isolated cells were suspended in DMEM supplemented with fetal bovine serum (FBS, 10%, heat inactivated), penicillin (100 IU/ml), streptomycin (100 μg/ml) and amphotericin B1 (0.25 μg/ml) (Antibiotic/Antimycotic) and plated on poly-L-lysine coated coverslips. Following 24-h incubation, the DMEM solution was replaced with Neurobasal media supplemented with B-27 (2%) and 0.5 mM L-glutamine. Cells were used after 10–21 days *in vitro*. All procedures were done in accordance with the regulations of the University of South Florida Institutional Animal Care and Use Committee.

### Calcium Imaging Measurements

Changes in intracellular Ca^2+^ concentrations, [Ca^2+^]_i_, were measured in isolated cortical neurons using fluorescent imaging techniques and the Ca^2+^ sensitive dye, fura-2. Cells were loaded using the membrane permeable ester form of fura-2, fura-2 acetoxymethyl ester (fura-2 AM), as previously described (DeHaven and Cuevas, 2004). Cells plated on coverslips were incubated for 1 h at room temperature in Neurobasal media with 4 μM fura-2 AM and 0.4% dimethyl sulfoxide (DMSO). The coverslips were then washed in fura-2 AM free physiological saline solution (PSS) prior to experiments being performed. Cells (20–60 per field of view), visualized using a 40x Achroplan objective (Zeiss Microscopy, White Plains, N.Y.), were alternately illuminated with 340 and 380 nm light at 0.8 Hz (Lambda DG-4, Sutter Instruments, Novato CA) and fluorescent emissions at 510 nm were collected using a Sensicam digital CCD camera (Cooke Corp., Auburn Hills, MI).

### Sodium Imaging Measurements

Changes in intracellular Na^+^ concentrations were measured in isolated cortical neurons using fluorescent imaging techniques and the Na^+^ sensitive dye, SBFI. Cells were loaded using the membrane permeable ester form of SBFI, SBFI acetoxymethyl ester (SBFI-2 AM). Cells plated on coverslips were incubated for 2 h at room temperature in Neurobasal media with 10 μM SBFI AM, 0.4% DMSO, 0.5% bovine serum albumin (BSA) and 0.1% pluronic. The coverslips were then washed in SBFI AM free physiological saline solution (PSS) prior to experiments being performed. The same microscope, objective, filter set, light source and camera as used for Ca^2+^ imaging were used. Images were acquired at 0.3 Hz.

### Electrophysiological Measurements

Membrane currents were recorded using the conventional and perforated, whole-cell patch clamp configurations as previously described (Cuevas, Harper, Trequattrini, and Adams, 1997). The conventional (dialyzing configuration) was only used to record voltage-gated sodium channel currents to prevent space-clamp. Briefly, glass coverslips plated with neurons were transferred to a recording chamber and continuously perfused with external solution at a rate of 350 μL/min. Patch electrodes were pulled from thin-walled borosilicate glass (World Precision Instruments Inc., Sarasota, FL) using a Sutter Instruments P-87 pipette puller (Novato, CA) and had resistances of 2.0–3.0 MΩ for conventional whole-cell patches and 1.0–1.5 MΩ for perforated patches. Access resistances (R_s_) were monitored throughout experiments for stable values ≤30 MΩ and were compensated at 40% (lag, 10 µs). When using the perforated-patch whole-cell configuration, electrical access was achieved with a pipette solution containing amphotericin B (Rae, Cooper, Gates, and Watsky, 1991). An amphotericin B stock solution (60 mg/ml in DMSO) was made fresh daily and diluted to 240 µg/ml (0.4% DMSO) in pipette solution immediately prior to use. To prevent current rundown when using the conventional whole-cell configuration, 3 mM ATP (disodium) was added to the internal solution. To isolate calcium currents, intracellular potassium was replaced with cesium, 70 mM external sodium was replaced with tetraethylammonium (TEA) and 2 μM tetrodotoxin (TTX) was added to the bathing solution. Perforated-patches were used to prevent clamping the intracellular calcium concentration by the pipet solution. To activate voltage-gated calcium currents, cells were held at −70 mV and stepped from −60 to +40 mV for 500 msec in 10 mV increments. To isolate sodium currents, intracellular potassium was replaced with cesium and 70 mM external sodium was replaced with TEA. 5 mM BaCl_2_ and 400 μM CdCl_2_ were added to a Ca^2+^-free bathing solution. The internal solution contained 10 mM NaCl (replacing 10 mM KCl), 5 mM TEA, 5 mM MgCl_2_ and 1 mM ethylene glycol tetraacetic acid (EGTA), requiring the use of conventional whole-cell patches to allow diffusion into the clamped cells. To activate voltage-gated sodium currents, cells were held at −70 mV and stepped from −50 to +5 mV for 50 msec in 5 mV increments. All membrane currents were amplified and filtered at 5 kHz with an Axon 200 amplifier, digitized at 10 kHz with a Digidata 1322A, and acquired using Clampex 10 (Axon) software. Currents were leak subtracted using the Clampex P/4 protocol.

### Solutions and Reagents

The control bath solution for all experiments was PSS, which contained (in mM): 140 NaCl, 5.4 KCl, 1.3 CaCl_2_, 1.0 MgCl_2_, 20 glucose, and 25 4-(2-hydroxyethyl)-1-piperazineethanesulfonic acid (HEPES) (pH to 7.4 with NaOH). Acidosis was produced using this solution adjusted to pH 6.0. Ischemia-acidosis was produced using glucose-free PSS with 4 mM sodium azide added and adjusting the solution to pH 6.0. The control pipette solution consisted of (in mM): 75 K_2_SO_4_, 55 KCl, 5 MgSO_4_, and 10 HEPES (titrated to pH 7.2 with N-methyl-d-glucamine). External solutions were applied using a rapid application system as previously described (Cuevas and Berg, 1998). To account for any rundown of responses, a paired protocol was used wherein cells were washed for 20 min between an initial control 2 min ischemia-acidosis application and the second 2 min ischemia-acidosis application in the absence or presence of bumetanide or ethacrynic acid, with second responses being normalized to the first responses (Katnik et al., 2006; Herrera et al., 2008). Bumetanide and ethacrynic acid were applied for 10 min prior to second ischemia-acidosis activations.

### Materials

All chemicals used in this investigation were of analytical grade. The following reagents were used: TEA (Acros Organics, Waltham, MA); TTX (Alomone Labs, Jerusalem, Israel); Antibiotic/Antimycotic, BSA, DMEM, DMSO, FBS (Fisher Scientific, Fair Lawn, NJ); B-27, Fura-2 AM, Neurobasal (Life Technologies, Carlsbad, CA); Ethacrynic Acid (MP Biomedicals, LLC, Solon, OH); and Bumetanide, EGTA, HEPES, L-Glutamine, Poly-L-Lysine (Sigma-Aldrich, St. Louis, MO).

### Data Analysis

Imaging data files were collected with SlideBook 4.02 (Intelligent Imaging Innovations, Inc.). Fluorescence emission intensities of individual fluorescent cells, measured using circular regions of interest (ROIs) placed over cell bodies, were collected as functions of time. For calcium imaging experiments, emission intensities were exported to SigmaPlot11, ratioed (R_340_/R_380_) and converted to [Ca^2+^]_i_ using the Grynkewicz equation with constant parameters R_min_, R_max_ and *β* determined using calibration solutions containing fura-2 salt. Differences in the values of these parameters from those obtained using an *in situ* protocol are controlled for by normalizing responses in the same cell. However, since [Ca^2+^]_i_ is not a linear function of R_340_/R_380_, this calculation is necessary to preserve the dynamic changes produced in the cell. For sodium imaging experiments, because the emission intensity of SBFI excited by 340 nm light is highly pH sensitive (Diarra, Sheldon, and Church, 2001), changes in [Na^+^]_i_ were depicted as changes in R_SBFI_, the emission intensity produced by 340 nm excitation of the cell at time 0 when pH = 7.4, I_340_(0), divided by the emission intensity of the cell excited by 380 nm light as a function of time, I_380_(t) (R_SBFI_ = I_340_(0)/I_380_(t)). Analyses of [Ca^2+^]_i_, R_SBFI_ and electrophysiological recordings were performed using Clampfit 10.5 (Axon Instruments). Statistical analysis was conducted using SigmaPlot 11 and SigmaStat 3 software (Systat Software, Inc.). Statistical differences were determined using paired and unpaired t-tests for within group and between group experiments, respectively, and were considered significant if *p* < 0.05. For multiple group comparisons 1- and 2-way ANOVAs, with or without repeat measures, were used, as appropriate. When significant differences were determined with an ANOVA, post-hoc analyses were conducted using a Dunn Test to determine differences between individual groups.

## Results

**FIGURE 1:**
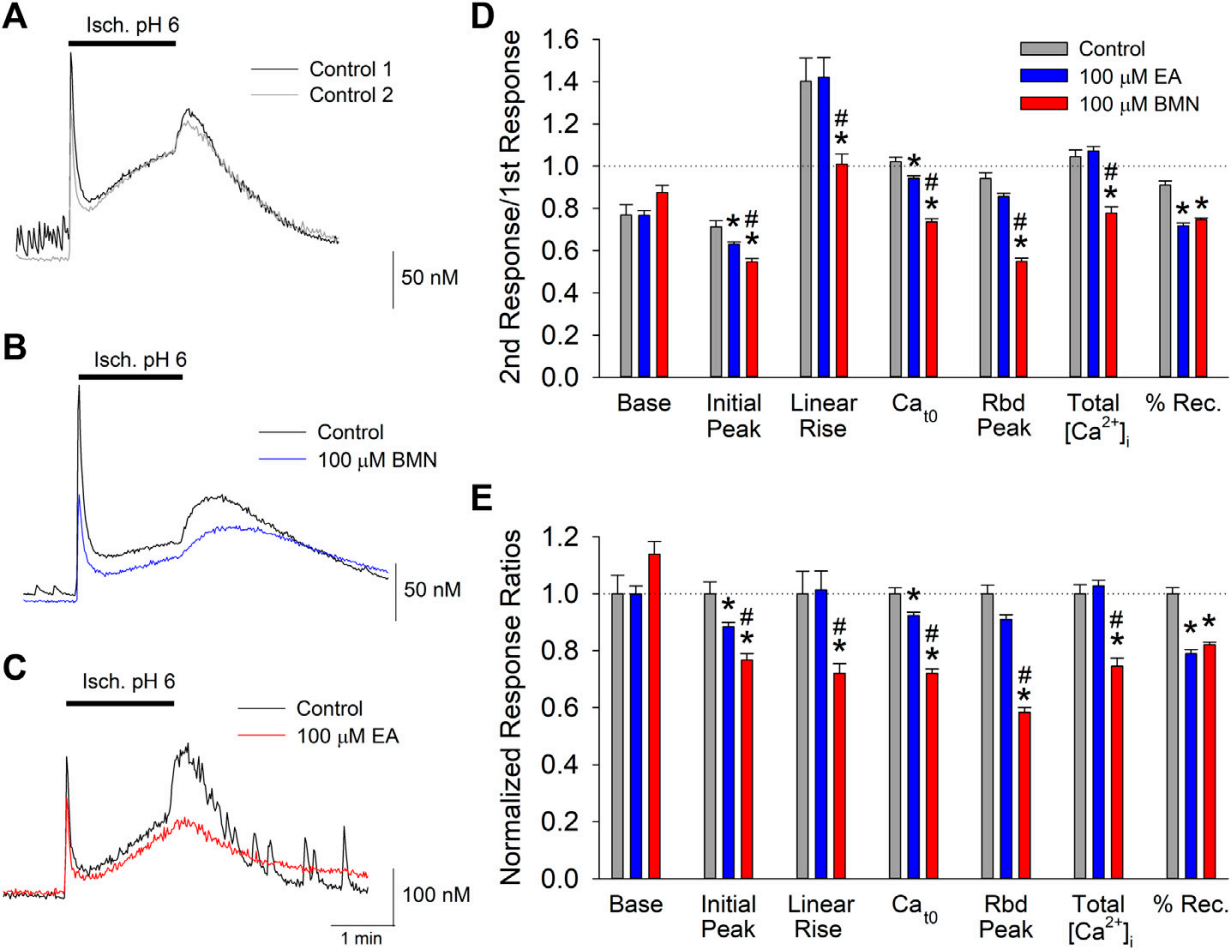
Ischemia-acidosis evoked [Ca^2+^]_i_ increases in cultured rat cortical neurons are inhibited by bumetanide and ethacrynic acid. A, Representative traces of [Ca^2+^]_i_ as a function of time recorded from a single neuron in response to an initial 2-min ischemia-acidosis insult (Isc. pH 6; Control 1, black trace) and a second application of the Isc. pH 6 solution following a 20-min recovery period (Control 2, gray trace). Representative traces of [Ca^2+^]_i_ recorded from two neurons using the same protocol as in **(A)**, in the absence and presence of 100 μM bumetanide [**(B)**; Control, black trace; 100 μM BMN, blue trace] or 100 μM ethacrynic acid [**(C)**; Control, black trace, 100 μM EA, red trace]. **(D)**, Relative mean [Ca^2+^]_i_ (±SEM) responses obtained using the paired protocol with the second response, in the absence (Control, *n* = 59) and presence of 100 μM bumetanide (BMN, *n* = 156) or 100 μM Ethacrynic Acid (EA, *n* = 140), normalized to the first control response. Peak [Ca^2+^]_i_ increase was measured immediately after application of the ischemia-acidosis solution. Sustained [Ca^2+^]_i_ was measured just prior to washout of the Isc. pH 6 solution. Rebound [Ca^2+^]_i_ was the maximum [Ca^2+^]_i_ measured following washout of Isc. pH 6 solution. The percent recovery (%Rec) was calculated as the final [Ca^2+^]_i_ normalized to the maximum [Ca^2+^]_i_ after the initial transient peak ([final-baseline]/[rebound peak-baseline]). Total [Ca^2+^]_i_ increases were calculated by integrating beneath the traces of [Ca^2+^]_i_ vs. time. **(E)**, Same data in **(D)** with ratios normalized to Control/Control ratios. Asterisks denote significant difference from Control (*p* < 0.05) and pound symbols indicate significant difference between BMN and EA (*p* < 0.05). Bars above **(A)**, **(B)** and **(C)** indicate duration of ischemia-acidosis (Isc. pH 6.0).

Our laboratory has shown that the concurrence of ischemia and acidosis, which occurs during stroke, results in a synergistic [Ca^2+^]_i_ overload in neurons and concomitant cell death (Mari et al., 2010). Given that NKCC1 has been implicated in [Ca^2+^]_i_ overload during reperfusion following oxygen-glucose deprivation (X. Chen et al., 2008), we examined the effects of the NKCC1 inhibitors, bumetanide and ethacrynic acid, on the [Ca^2+^]_i_ burden produced by simultaneous ischemia and acidosis in neurons. Consistent with previous studies, *in vitro* ischemia-acidosis (Isc. pH 6) induced a rapid, transient increase in [Ca^2+^]_i_ in cortical neurons followed by a slow steady rise in the continued presence of the Isc. pH 6 solution. Upon washout of the solution, the [Ca^2+^]_i_ rebounded to a second, slower decaying peak, before returning to near baseline levels (Figure 1A). Application of 100 μM bumetanide (Figure 1B, blue trace) or 100 μM ethacrynic acid (Figure 1C, red trace) resulted in a reduction in [Ca^2+^]_i_ elevations produced by ischemia-acidosis. Additionally, [Ca^2+^]_i_ recovered more slowly in the presence of these inhibitors. Combined data from identical experiments showed both bumetanide and ethacrynic acid reduced the initial peak increases and the sustained levels of [Ca^2+^]_i_ triggered by ischemia-acidosis. However, ethacrynic acid produced a statistically greater reduction in both of these [Ca^2+^]_i_ increases relative to bumetanide. Furthermore, ethacrynic acid, but not bumetanide, reduced the peak rebound increase in [Ca^2+^]_i_ observed upon washout of ischemia-acidosis (Figures 1D,E). While the loop diuretics inhibited the transient increases, they also decreased the rate of recovery from the elevated [Ca^2+^]_i_, resulting in an elevated [Ca^2+^]_i_ at the end of the recording period (9 min) relative to Control (Figures 1D,E). The total increase in [Ca^2+^]_i_ induced by ischemia-acidosis, as measured by integrating under the traces (Total [Ca^2+^]_i_), is dependent on the transient increases and as well as the degree of recovery during the recording period. For cells incubated in bumetanide, the decreased elevations in [Ca^2+^]_i_ occurring during ischemia-acidosis were offset by the reduced recovery during washout. Thus, bumetanide failed to reduce net [Ca^2+^]_i_ increases relative to Control. In contrast, ethacrynic acid, which produced a similar degree of inhibition of recovery as bumetanide, but a greater block of initial peak, sustained and rebound peak [Ca^2+^]_i_ than bumetanide, reduced the net [Ca^2+^]_i_ increase produced by ischemia-acidosis (Figures 1D,E).

**FIGURE 2:**
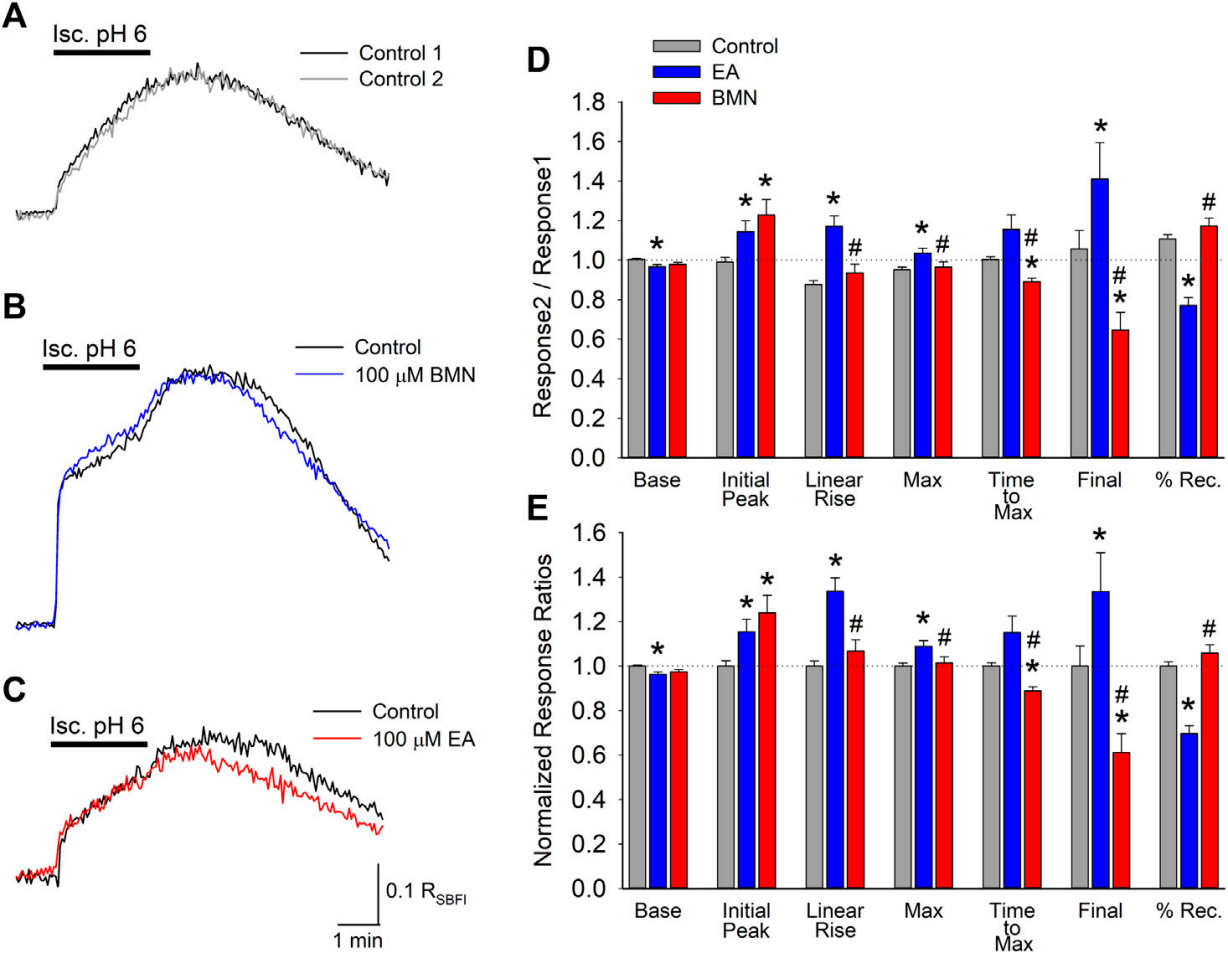
Increases in [Na^+^]_i_, depicted by R_SBFI_, evoked by concurrent ischemia and acidosis are not inhibited by NKCC1 inhibitors. **(A)**, Representative traces of R_SBFI_ as a function of time recorded from a single neuron during two, 2-min ischemia-acidosis insults with a 20 min recovery period in between. **(B)**, Representative traces of R_SBFI_ as a function of time recorded from a single neuron in response to two, 2-min ischemia-acidosis applications, first in the absence (Control, black trace) and 20 min later in the presence of 100 μM bumetanide (100 μM BMN, blue trace). **(C)**, Representative traces of R_SBFI_ as a function of time recorded from a single neuron in response to two, 2-min ischemia-acidosis applications, first in the absence (Control, black trace) and 20 min later in the presence of 100 μM ethacrynic acid (100 μM EA, red trace). **(D)**, Relative mean R_SBFI_ (±SEM) measured in the absence (Control) and presence of 100 μM bumetanide (100 μM BMN, *n* = 108) or 100 μM ethacrynic acid (100 μM EA, *n* = 49). Responses were normalized to control responses in the same cells. Baseline and Initial Peak represent R_SBFI_ values measured immediately prior to and 15 s after initiation of ischemia-acidosis. Max Peak represents highest R_SBFI_ measured during ischemia + acidosis. Recovery was calculated as (final-baseline)/(maximum-baseline). **(E)**, Same data presented in **(D)** but normalized to the Response2/Response1 ratio for Control. Asterisks denote significant difference from Control (*p* < 0.05) and pound symbol indicates significant difference between BMN and EA (*p* < 0.05). Bars above **(A)** and **(B)** indicate duration of ischemia + acidosis (Isc. pH 6.0).

In addition to affecting [Ca^2+^]_i_, NKCC1 has been implicated in elevations in [Na^+^]_i_ during post-ischemia reperfusion (X. Chen et al., 2008). This [Na^+^]_i_ overload causes cellular edema and promotes cell death. Thus, it was of interest to determine if NKCC1 plays a significant role in intracellular Na^+^ homeostasis during ischemia-acidosis and during reperfusion. SBFI loaded cultured cortical neurons were imaged to determine the effects of ischemia-acidosis on intracellular Na^+^ by monitoring R_SBFI_. Ischemia-acidosis was found to produce an immediate rapid rise in [Na^+^]_i_ followed by a slow steady increase that persisted for approximately 1 min following washout of the ischemia-acidosis solution before [Na^+^]_i_ returned towards baseline (Figure 2A). Application of either 100 μM bumetanide or 100 μM ethacrynic acid failed to significantly alter increases in [Na^+^]_i_ produced by ischemia-acidosis, but both effected recovery from these [Na^+^]_i_ elevations (Figures 2B,C). Compiled data showed that basal [Na^+^]_i_ was not affected by either bumetanide or ethacrynic acid (Figures 2D,E). Similarly, neither the initial peak [Na^+^]_i_ nor the maximum peak [Na^+^]_i_ were significantly affected by the NKCC1 inhibitors (Figures 2D,E). However, opposite effects on the recovery from these induced [Na^+^]_i_ increases were noted for the two NKCC1 inhibitors, with bumetanide slowing down recovery and ethacrynic acid accelerating return to baseline [Na^+^]_i_ (Figure 2D).

**FIGURE 3:**
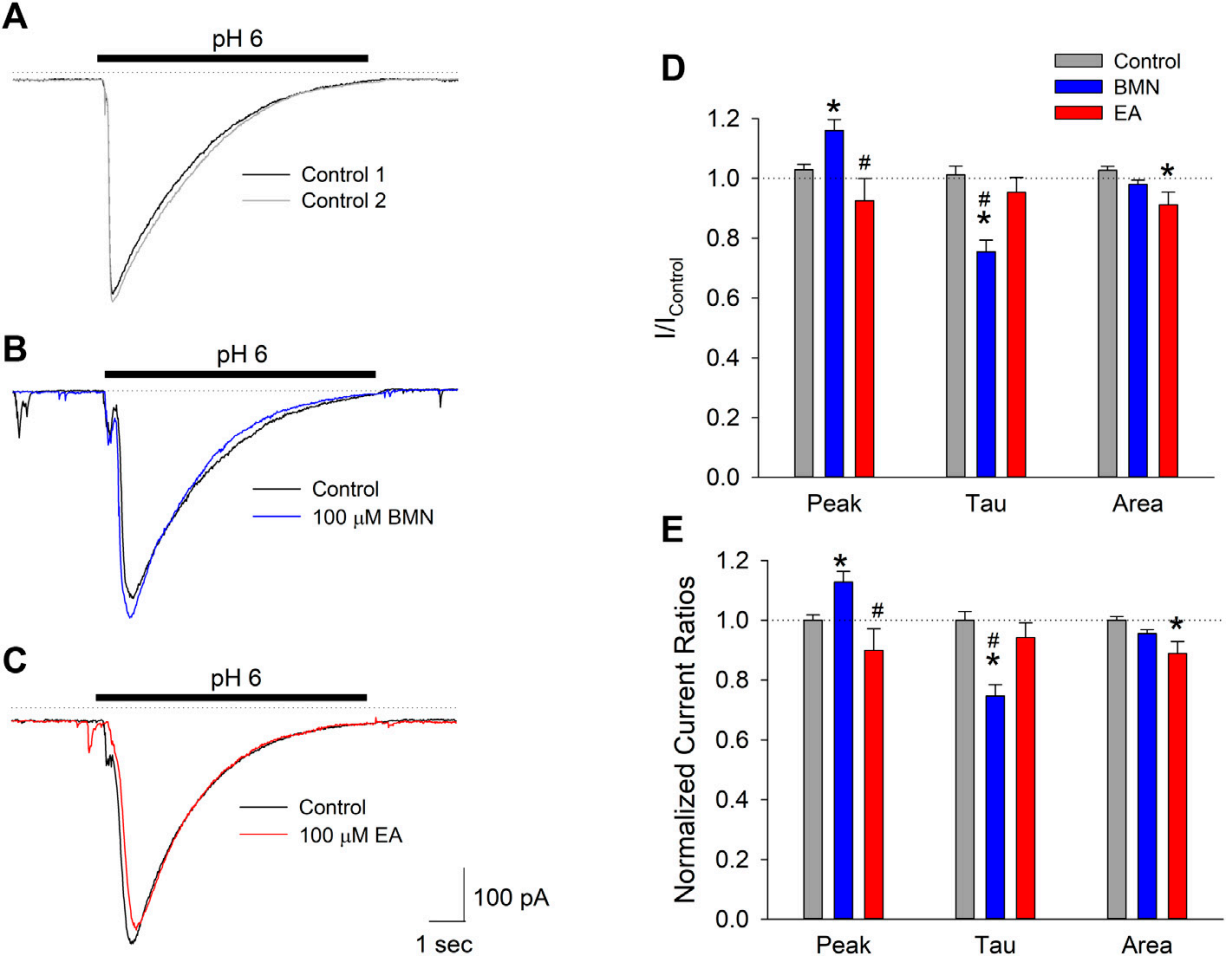
Peak inward ASIC-mediated currents are not inhibited by bumetanide or ethacrynic acid. **(A)**, Representative traces of inward currents activated by two 10 s applications of acidic solution (pH 6.0) onto a single cell voltage clamped at −70 mV. **(B)**, Representative traces of inward currents activated by 10 s applications of PSS at pH 6.0 onto a single cell voltage-clamped at −70 mV in the absence (Control, black trace) and presence of 100 μM bumetanide (100 μM BMN, blue trace). **(C)**, Representative traces of inward currents activated by 10 s applications of PSS at pH 6.0 onto a single cell voltage-clamped at −70 mV in the absence (Control, black trace) and presence of 100 μM ethacrynic acid (100 μM EA, red trace). **(D)**, Mean peak inward currents, rates of current decay (*τ*) and net current (area under current trace) (±SEM) measured in the absence (Control, *n* = 16) and presence of 100 μM bumetanide (BMN, *n* = 6) and 100 μM ethacrynic acid (EA, *n* = 10) normalized to initial control responses (absence of drugs). **(E)**, Data in **(D**) normalized to Response2/Response1 ratio for Control. Asterisks denote significant difference from Control (*p* < 0.05) and pound symbols indicate significant difference between BMN and EA (*p* < 0.05). Bars above **(A)**, **(B)**, and **(C)** indicate duration of low pH application (pH 6.0). In all experiments, second low pH solution application was carried out after a 5 min washout of the initial acid stimulation.

The fact the two NKCC1 antagonists, bumetanide and ethacrynic acid, failed to block elevations in [Na^+^]_i_ observed following ischemia-acidosis suggests that NKCC1 is not the site of action responsible for the mitigation of [Ca^2+^]_i_ overload by these drugs under these conditions. Our laboratory has previously shown the initial, transient rise in [Ca^2+^]_i_ following ischemia-acidosis is dependent on activation of acid-sensing ion channels (ASIC) (Mari et al., 2010). Since this rise was BMN- and EA-sensitive, we examined the effects of these loop diuretics on ASIC-mediated whole-cell currents in isolated cortical neurons. Application of acidic PSS (pH 6.0) evoked transient inward currents in neurons voltage-clamped at −70 mV, consistent with ASIC activation (Figure 3A). Bumetanide (100 μM) was found to increase peak current amplitudes by 13% and decay times by 25% which resulted in no significant difference in the net inward current induced by 10 s acidosis compared to control (Figures 3B,D,E). In contrast, while ethacrynic acid (100 μM) did not statistically alter the peak inward current amplitude or the time constant of its decay, the small apparent decrease in both resulted in a 10% block of the net inward current produced by 10 s acidosis (Figure 3C,D,E). These results, however, are not consistent with the 10 and 20% blocks of initial [Ca^2+^]_i_ increases by BMN and EA, respectively (see Figure 1), which suggests that these drugs are modulating Ca^2+^ influx pathways distinct from ASIC.

**FIGURE 4:**
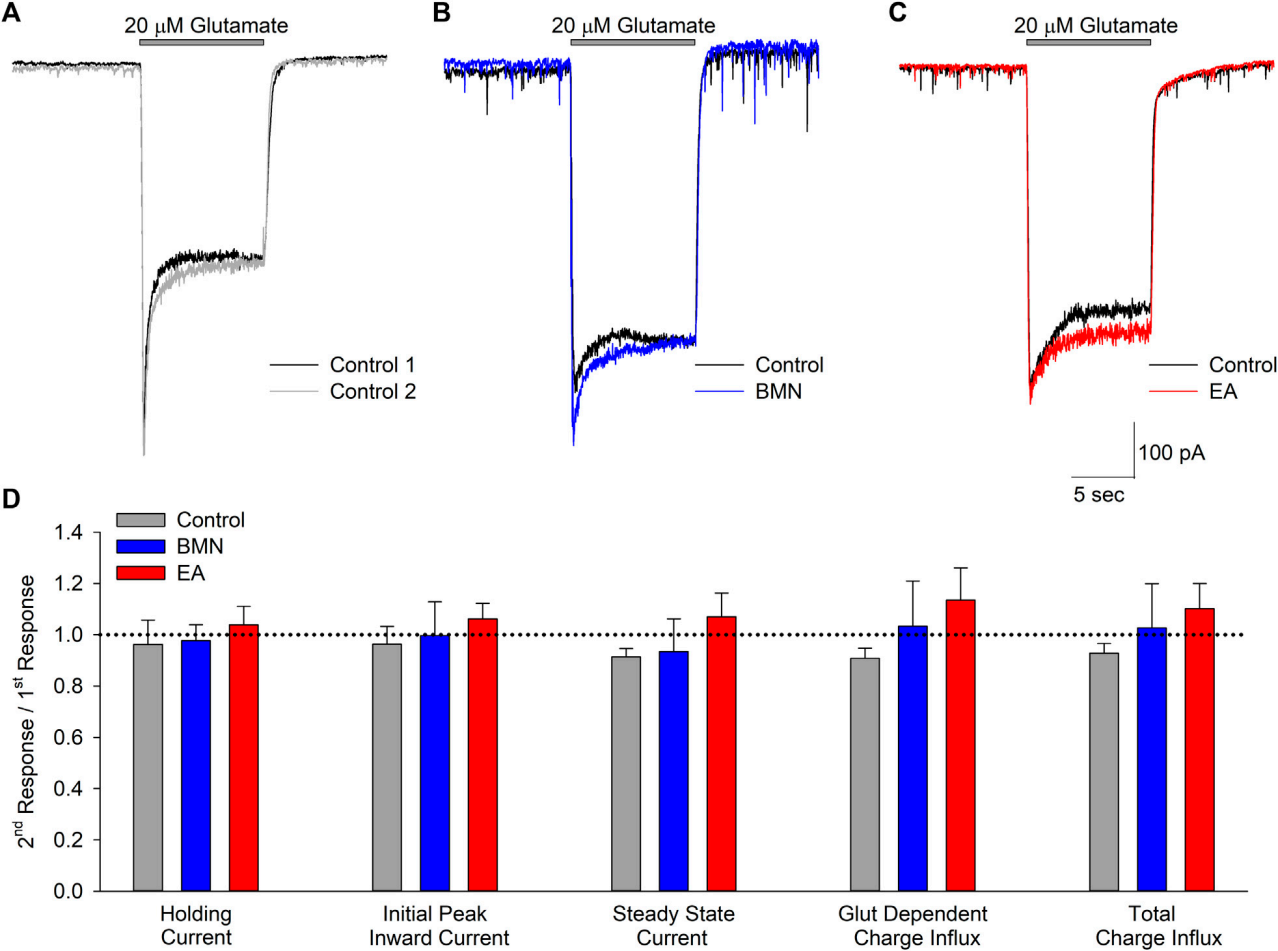
Glutamate activated inward currents are not inhibited by bumetanide or ethacrynic acid. **(A)**, Representative traces of whole-cell currents recorded from a single neuron voltage clamped at −70 mV in response to two 10 s applications of 20 μM glutamate. **(B)**, Representative traces of whole-cell currents recorded from a single neuron voltage clamped at −70 mV in response to a 10 s application of 20 μM glutamate in the absence (Control, black trace) and presence of 100 μM bumetanide (100 μM BMN, blue trace). **(C)**, Representative traces of whole cell currents recorded from a single neuron voltage clamped at −70 mV in response to a 10 s application of 20 μM glutamate in the absence (Control, black trace) and presence of 100 μM ethacrynic acid (100 μM EA, red trace). **(D)**, Bar graph of compiled data from experiments identical to those in A (*n* = 5), B (*n* = 4) and C (*n* = 4). Data are shown as the ratio of second responses to first responses in the absence (Control) and presence of drug (BMN or EA). Data shown are mean response ratios (±SEM) of holding current, initial peak and steady state (measured at the end of the 10 s application) inward currents, and charge influx during glutamate application (Glut Dependent) and throughout the recording (Total). No significant differences were noted for any of the parameters measured (*p* > 0.12 for all). Bars above traces in A-C denote times of glutamate application.

Activation of NMDA receptors has also been shown to occur during ischemia-acidosis and produces increases in neuronal [Ca^2+^]_i_ (Mari et al., 2010). To determine if bumetanide and/or ethacrynic acid inhibit ionotropic glutamatergic currents, whole cell currents were recorded in neurons voltage clamped at -70 mV and perfused with 20 μM glutamate in the absence and presence of the two drugs (Figures 4A–C). The NCCK1 inhibitors did not produce appreciable changes in ionotropic glutamatergic responses (Figures 4B,C). Figure 4D shows bar graph of mean peak, steady-state and net inward currents (Charge Influx) recorded from multiple neurons during 10 s glutamate applications. Neither 100 μM bumetanide nor 100 μM ethacrynic acid significantly inhibited any of the components of the glutamate-evoked currents (Figures 4D).

**FIGURE 5:**
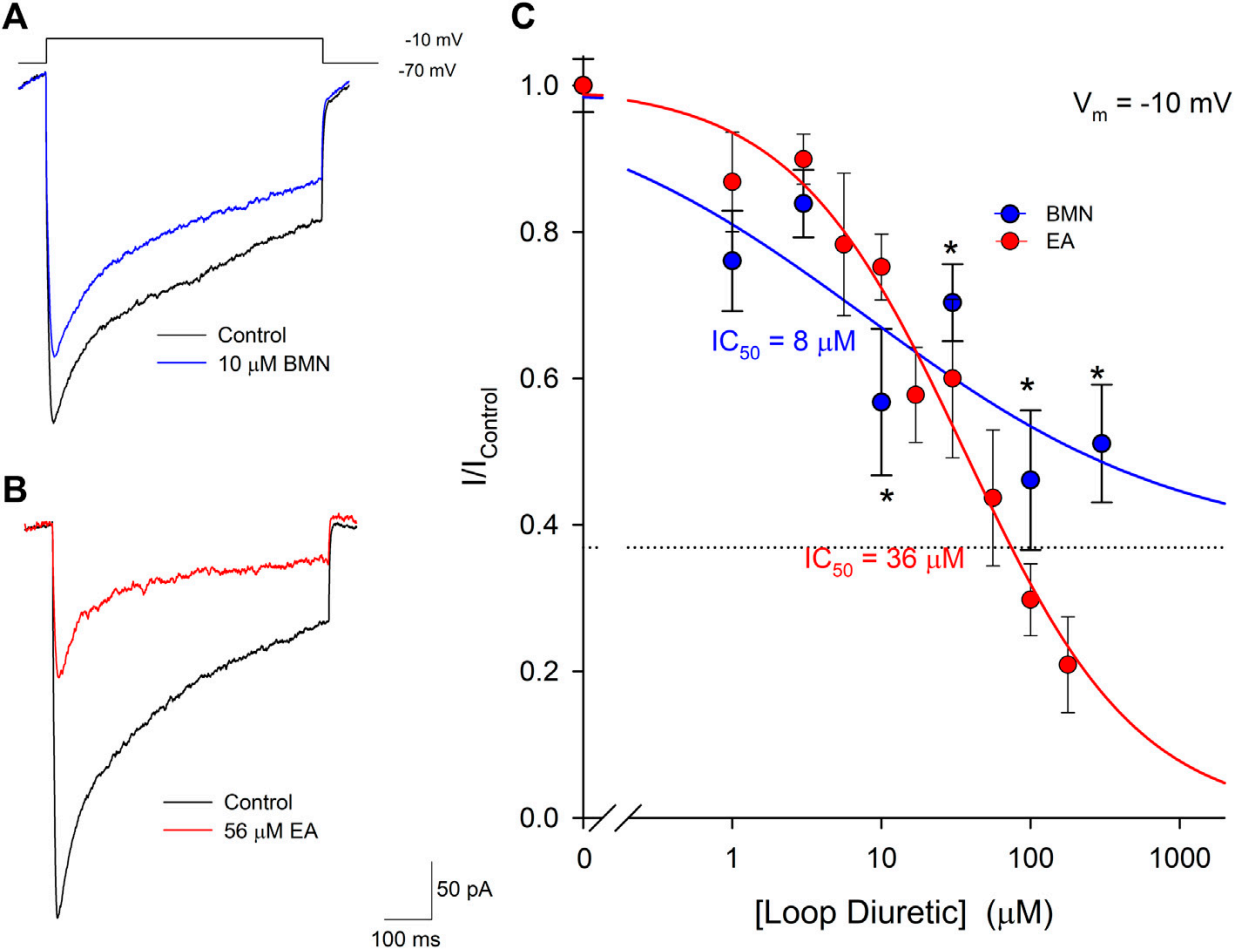
Bumetanide and ethacrynic acid inhibit voltage-gated calcium channels in a dose-dependent manner. **(A)**, Representative traces of whole-cell VGCC currents recorded from a single neuron in response to a 500 msec step from a holding potential of −70 mV to −10 mV in the absence (Control, black trace) and presence of 10 μM bumetanide (10 μM BMN, blue trace). **(B)**, Representative traces of whole-cell VGCC currents recorded from a single neuron in response to a 500 msec step from a holding potential of -70 mV to −10 mV in the absence (Control, black trace) and presence of 56 μM ethacrynic acid (56 μM EA, red trace). **(C)**, Concentration-response relationships of relative peak inward VGCC currents (mean ± SEM) activated by voltage steps from −70 mV to −10 mV. Currents were normalized to control peak currents measured in the absence of bumetanide or ethacrynic acid. Data points (bumetanide blue circles; ethacrynic acid red circles) were best fit (bumetanide blue line, ethacrynic acid red line) using a single-site Langmuir-Hill equation with an asymptotic minimum (dashed line for BMN). *n* > 7 for each data point. Asterisks denote significant difference from Control (*p* < 0.05).

The inability of bumetanide to inhibit acidosis- and glutamate-evoked inward currents in cultured cortical neurons raises the possibility that the reduction of ischemia-acidosis induced [Ca^2+^]_i_ increases produced by this NCCK1 inhibitor might be due to block of Ca^2+^ influx through voltage-gated Ca^2+^ channels (VGCC). Previous studies in our laboratory have shown VGCC are activated downstream of ASIC following ischemia-acidosis (Mari et al., 2010). Neurons were voltage-clamped at -70 mV and depolarizing membrane pulses applied to -10 mV, in the presence of TTX and TEA, to isolate currents through VGCC. Figure 5A shows representative membrane currents recorded from a single neuron in response to membrane depolarizations in the absence (Control) and presence of 10 μM bumetanide (BMN). At this concentration, bumetanide decreased the peak current amplitude by approximately 40%. Figure 5B shows representative membrane currents recorded from a single neuron in response to membrane depolarizations in the absence (Control) and presence of 56 μM ethacrynic acid (EA). In this cell, 56 μM ethacrynic acid produced an approximately 60% reduction in the VGCC-mediated current. Using identical methods, concentration-response relationships were constructed for bumetanide and ethacrynic acid inhibition of VGCC-mediated currents (Figure 5C). Data points were best fit with a single-site Langmuir-Hill equation (Cuevas and Adams, 1997) with half-maximal inhibitions (IC_50_), Hill coefficients and non-reducible current (asymptotic minimum) values of 8 μM, 0.41 and 0.37, respectively for BMN (blue circles and line) and 36 μM, 0.75 and 0.0 for EA (red circles and line) (Figure 5C). Thus, ethacrynic acid has lower affinity for the channel, but greater efficacy than bumetanide for blocking VGCCs in cortical neurons.

**FIGURE 6:**
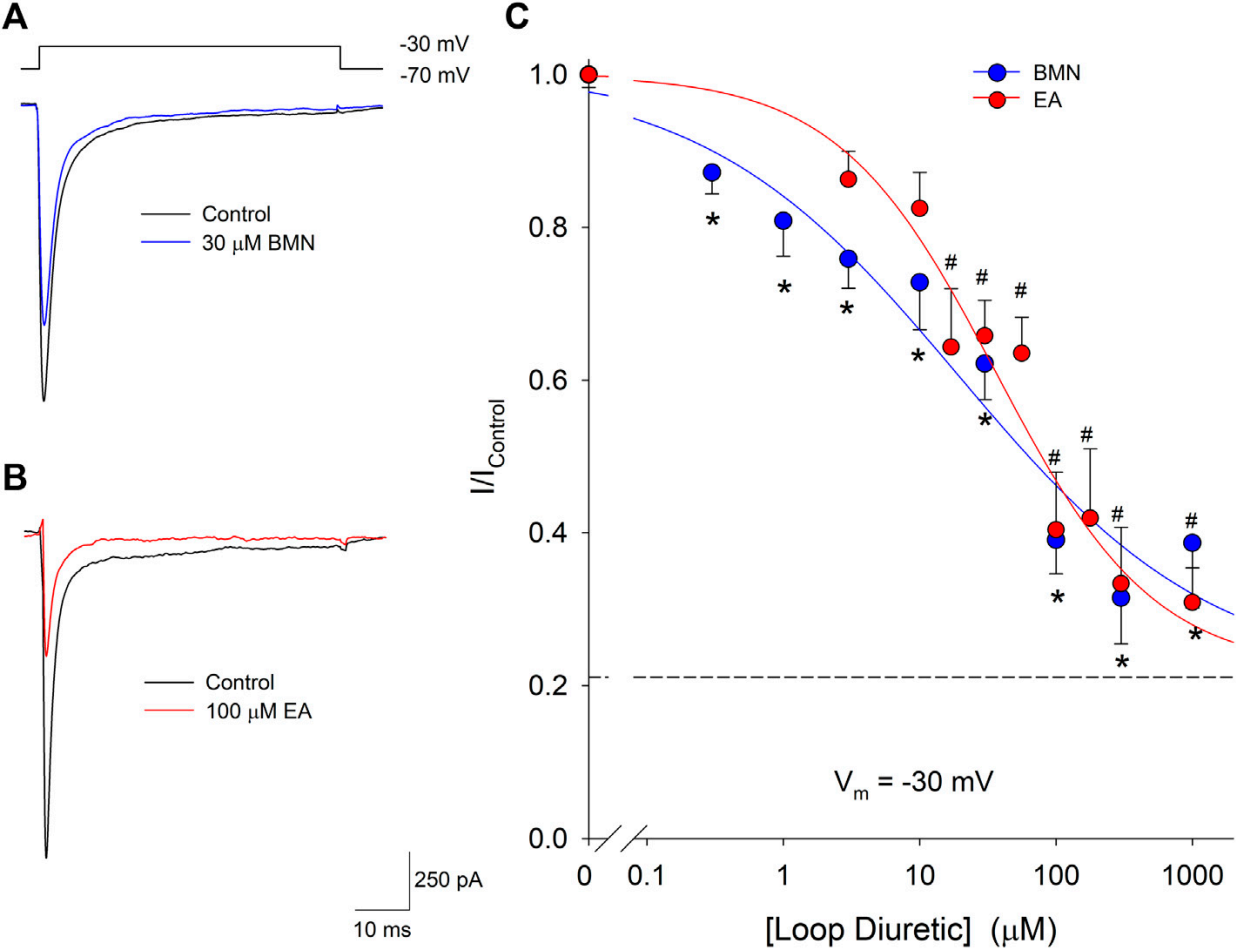
Bumetanide and ethacrynic acid inhibit voltage-gated sodium channels in a dose-dependent manner. **(A)**, Representative traces of whole-cell VGSC currents recorded from a single neuron in response to 50 msec steps from a holding potential of −70 mV to −30 mV in the absence (Control, black trace) and presence of 30 μM Bumetanide (30 μM BMN, blue trace). **(B)**, Representative traces of whole-cell VGSC currents recorded from a single neuron in response to 50 msec steps from a holding potential of −70 mV to −30 mV in the absence (Control, black trace) and presence of 100 μM ethacrynic acid (100 μM EA, red trace). **(C)**, Concentration-response relationship of relative peak inward VGSC currents (mean ± SEM) elicited by voltage steps from −70 to −30 mV. Currents were normalized to control peak currents measured in the absence of bumetanide or ethacrynic acid. Data points (bumetanide blue circles; ethacrynic acid red circles) were best fit (bumetanide blue line, ethacrynic acid red line) to a single-site Langmuir-Hill equation with an asymptotic minimum (dashed line). *n* > 15 for all bumetanide data points and n > 6 for all ethacrynic acid data points. Asterisks and pound symbols denote significant difference from Control for BMN and EA, respectively (*p* < 0.05). Neurons for these experiments were electrically accessed using the conventional (dialyzing) whole-cell patch clamp recording configuration.

Our laboratory has shown that elevations of [Ca^2+^]_i_ triggered by acidosis can also be reduced via the inhibition of voltage-gate Na^+^ channels (VGSC). Furthermore, blocking of VGSC has been shown to lessen neuronal [Ca^2+^]_i_ increases observed after oxygen-glucose deprivation (LoPachin, Gaughan, Lehning, Weber, and Taylor, 2001; Herrera et al., 2008). Thus, we examined if the loop diuretics, bumetanide and ethacrynic acid, also effected VGSC in cortical neurons. Inward VGSC currents were activated by stepping voltage clamped neurons from −70 mV to −30 mV in the presence of TEA, Ba^2+^ and Cd^2+^. Representative VGSC currents recorded from a single neuron are shown in Figure 6A and demonstrate BMN inhibit VGSC. Similarly, VGSC currents were reduced by micromolar concentrations of EA (Figure 6B). Concentration-response relationships were constructed using measurements from 86 neurons and concentrations of bumetanide ranging from 0.3 to 1,000 μM and on 46 neurons using concentrations of ethacrynic acid ranging from 3 to 1,000 μM. Figure 6C shows the data points were best fit with a single-site Langmuir-Hill equation with IC_50_, Hill coefficient and non-reducible current (asymptotic minimum) values of 19 μM, 0.46 and 0.21, respectively for BMN (blue circles and line) and 36 μM, 0.72 and 0.22, respectively for EA (red circles and line). These results indicate nearly 20% of the VGSC current is BMN and EA-insensitive.

It should also be noted that the blocks of these voltage-gated currents by the loop diuretics were not voltage dependent. There were no observable shifts in the voltages for maximal current amplitude (Supplementary Figure S1) in the presence of half-maximal concentrations of BMN or EA compared to control.

**FIGURE 7:**
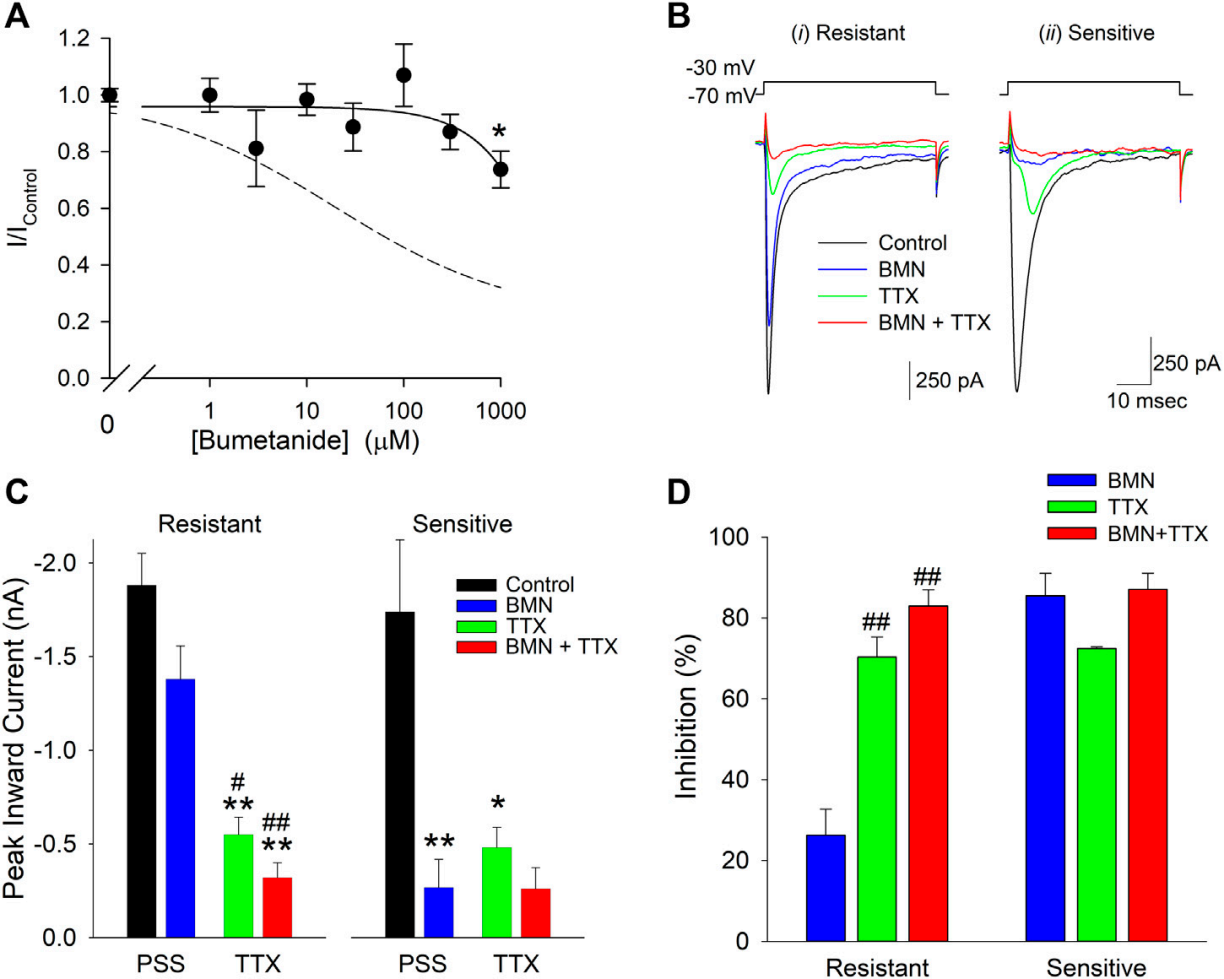
Sensitivity to inhibition by bumetanide distinguishes two populations of voltage-gated sodium channels in cultured neurons. **(A)**, Normalized peak inward VGSC currents (mean ± SEM) as a function of bumetanide concentration recorded from neurons with VGSC resistant to bumetanide blockade (*n* > 7). Currents were activated by voltage steps from a holding potential of −70 mV to −30 mV and normalized to control peak currents measured in the absence of bumetanide at the indicated concentrations. Data points were best fit (solid line) to a linear equation with a slope of −0.2 nM^−1^. Dotted line is the single-site Langmuir-Hill equation fit to the data from the bumetanide-sensitive neurons shown in Figure 6. **(B)**, Representative traces of whole-cell currents evoked by 50 msec voltage steps from a holding potential of −70 mV to −30 mV recorded from two different neurons, one with primarily VGSC currents resistant to bumetanide (*i*, Resistant) and one with VGSC sensitive to inhibition by bumetanide (*ii*, Sensitive). Currents were elicited from the neurons in the absence of drug (Control, black traces), and in the presence of 1 mM Bumetanide (BMN, blue traces), 100 nM TTX (TTX, green traces) and 1 mM Bumetanide +100 nM TTX (BMN + TTX, red traces). **(C)**, Peak inward VGSC currents (mean ± SEM) measured from 4 bumetanide-resistant (Resistant, left panel) and 6 bumetanide-sensitive (Sensitive, right panel) neurons using the same protocol as **(B)**. A two-way ANOVA followed by a post-hoc Tukey Test indicates no significant interaction between BMN and TTX in the Resistant group (*p* = 0.336), but a significant interaction between TTX and BMN in the Sensitive group (*p* < 0.02). A one-way ANOVA followed by a post-hoc Tukey Test on the Resistant group indicates TTX and BMN + TTX are both significantly different from Control (*p* < 0.001) and BMN (*p* < 0.05 and 0.001, respectively) but not each other and BMN was not significantly different from Control (*p* = 0.079). **(D)**, Percent block of normalized peak VGSC currents (Percent block = (1-I/I_Control_)*100) calculated from the currents measured in **(B)** for cells Resistant (left panel) or Sensitive (right panel) to BMN. A one-way ANOVA indicates BMN produces a statistically significant lower block than TTX or TTX + BMN in resistant cells (*p* < 0.001). No significant differences were noted for block in the BMN-sensitive cells (*p* = 0.10). Asterisks indicate significant difference from Control, pound sign indicates significant difference from BMN determined by Two-Way ANOVA (*^,#^ *p* < 0.05: **^,##^ *p* < 0.001).

The 70% block of VGSC currents by maximal concentrations of bumetanide only occurred in a fraction of the neurons tested. Of the 103 cells used to measure bumetanide inhibition of VGSC currents, 31 were found to be inhibited by ∼20% by millimolar concentrations of bumetanide. A concentration-response relationship for VGSC currents in bumetanide-resistant neurons failed to exhibit any concentration-dependence of block (solid line), in contrast to the dose-response relationship observed in the bumetanide-sensitive neurons (dashed line) (Figure 7A). Figure 7B shows current traces from two different voltage clamped neurons stepped from a holding potential of −70 to −30 mV in the absence and presence of 1 mM bumetanide, 100 nM TTX and 1 mM BMN + 100 nM TTX. The current traces on the left are from a representative neuron resistant to bumetanide block, whereas the traces on the right are from a neuron sensitive to bumetanide block. The data from 10 cells exposed to this protocol were grouped according to bumetanide sensitivity and each group analyzed with a two-way ANOVA to determine if there were interactions between TTX and bumetanide (Figure 7C). For the bumetanide resistant currents, there was no interaction between the two compounds (*p* = 0.336) (Figure 7C), while, in bumetanide sensitive neurons there was a statistically significant interaction between the drugs, such that the response to bumetanide was dependent on the presence of TTX (*p* < 0.02) (Figure 7C). While both BMN and TTX reduced the VGSC current amplitude relative to control alone (*p* < 0.001 and *p* = 0.002, respectively), the combination BMN + TTX was not significantly different from either BMN (*p* = 0.981) or TTX (*p* = 0.49) alone (Figure 7C). To further facilitate comparison, we determined the percent block observed for each condition, BMN, TTX and BMN + TTX. Figure 7D shows the percent inhibition (mean ± SEM) observed for both bumetanide-resistant (left panel) and bumetanide-sensitive (right panel) neurons. In bumetanide-resistant neurons, 1 mM BMN inhibited VGSC currents by only ∼26%, while TTX and BMN + TTX inhibited the responses by over 70% (Figure 7D). In these neurons, there were statistically significant blocks produced by TTX and BMN + TTX. The block by BMN alone was significantly different from inhibition with TTX present (*p* < 0.001). In contrast, VGSC currents from the bumetanide-sensitive cells were all blocked by >70% by BMN, TTX and BMN + TTX (Figure 7C) and these inhibitions were not significantly different from each other.

## Discussion

The primary finding of this study is the loop diuretics bumetanide and ethacrynic acid inhibit voltage-gated Ca^2+^ and Na^+^ ion channels that contribute to intracellular Ca^2+^ dysregulation evoked by ischemia-acidosis in neurons. The IC_50_ values of BMN and EA for these channels are consistent with the concentrations of the loop diuretics required to inhibit the ischemia-acidosis evoked [Ca^2+^]_i_ overload. Neither loop diuretic significantly blocked acidosis- or glutamate-activated whole cell inward currents, suggesting that the inhibition of ischemia-acidosis induced [Ca^2+^]_i_ overload was not due to block of ASIC or glutamatergic ion channels. In addition, neither BMN nor EA significantly altered ischemia-acidosis induced increases in [Na^+^]_i_. Thus, the increase in [Na^+^]_i_ induced by ischemia-acidosis is not mediated by Na^+^ influx through either VGSCs or NKCC. These observations further suggest that loop diuretic suppression of [Ca^2+^]_i_ overload during ischemia-acidosis is not a downstream effect of mechanisms activated to preserve [Na^+^]_i_ homeostasis.

Our laboratory first showed ischemia and acidosis interact to produce a synergistic increase in neuronal [Ca^2+^]_i_ overload (Mari et al., 2010). While several ion channels, including acid-sensing ion channels, VGCC and NMDA receptors were all found to contribute to the increases in [Ca^2+^]_i_, use of specific blockers of these channels suggested additional molecular mechanisms were likely involved in the ischemia-acidosis evoked [Ca^2+^]_i_ dysregulation. Both bumetanide and ethacrynic acid inhibited multiple components of the ischemia-acidosis evoked [Ca^2+^]_i_ overload, including the initial transient increase, sustained levels and rebound peak increase in [Ca^2+^]_i_. This suggested bumetanide and ethacrynic acid are either inhibiting multiple proteins that cause this [Ca^2+^]_i_ overload or are affecting mechanisms that directly or indirectly influence [Ca^2+^]_i_ handling throughout the ischemia-acidosis event. Activation of ASIC, NMDA and VGCC all produce rapid increases in neuronal [Ca^2+^]_i_ and impact the initial rapid transient increase in [Ca^2+^]_i_ (Mari et al., 2010; Mari, Katnik, and Cuevas, 2015). However, neither bumetanide nor ethacrynic acid were found to appreciably block ASIC- or NMDA-mediated currents at concentrations as high as 100 μM. Thus, it is unlikely inhibition of these channels by the loop diuretics produces the reduction in ischemia-acidosis evoked synergistic increases in neuronal [Ca^2+^]_i_ overload. In contrast, both bumetanide and ethacrynic acid were found to inhibit VGCC.

These loop diuretics are inhibitors of the renal Na-K-Cl cotransporters, NKCC1 and NKCC2. Bumetanide has been shown to inhibit rat NKCC1 and NKCC2 with IC_50_ values of ∼6 μM (Hannaert, Alvarez-Guerra, Pirot, Nazaret, and Garay, 2002). The IC_50_ observed in the current study for bumetanide inhibition of voltage-gated Ca^2+^ channels, 8 μM, is nearly identical to that for NKCC1/NKCC2. However, while bumetanide at the highest concentrations (>50 μM) blocked nearly all of the activity of both NKCC1 and NKCC2 (Hannaert et al., 2002), over 40% of the VGCC current was resistant to 300 μM bumetanide. It remains to be determined if this bumetanide-resistant component represents a specific VGCC subtype or if the compound has low efficacy for VGCC in general. While the effects of loop diuretics on neuronal VGCC were previously unknown, bumetanide can inhibit VGCC in cardiac myocytes at low μM concentrations (Shimoni, 1991). Inhibition of VGCC in cardiomyocytes by bumetanide was found to be as high as 80% of the peak VGCC current and varied significantly from cell to cell (Shimoni, 1991).

Ethacrynic acid is structurally dissimilar to bumetanide and higher concentrations are required to inhibit both transporter subtypes. EA inhibits NKCC1 in avian erythrocytes and NKCC2 in canine renal epithelial cells with IC_50_ values of 180 and 20 μM, respectively, (Rugg, Simmons, and Tivey, 1986; Palfrey and Leung, 1993). Given the IC_50_ value for EA inhibition of VGCC is 36 μM, EA is a more potent inhibitor of VGCC than NKCC. While EA was less potent than bumetanide at inhibiting VGCC, EA exhibited greater efficacy for these channels, with 178 μM EA blocking ∼80% of the VGCC currents in the neurons. Unlike bumetanide, ethacrynic acid, which is structurally dissimilar and does not contain a sulfonamide substituent, has not been previously shown to modulate VGCC in any cell type.

Bumetanide blocked voltage-gated Na^+^ channels with an IC_50_ (19 μM) similar to its IC_50_ for NKCC1 and NKCC2. In contrast to observations of VGCC block by bumetanide in the current study, a population of cortical neurons expressed VGSC that were resistant to bumetanide block, even at the highest concentrations (1 mM). In both bumetanide-sensitive and bumetanide-insensitive cells, VGSC currents were blocked by >70% by TTX (100 nM). Bumetanide application did not have an additive effect with TTX, suggesting bumetanide specifically inhibits one of the TTX-sensitive channel subtypes expressed in cortical neurons. Cortical neurons express a variety of TTX-sensitive VGSC, including NaV1.1, NaV1.2, NaV1.3, NaV1.6 and NaV1.7 and the TTX-insensitive NaV1.9 (Jeong et al., 2000; de Lera Ruiz and Kraus, 2015; Rubinstein et al., 2016). Thus, bumetanide does not appear to affect NaV1.9. The specific TTX-sensitive NaV subtype affected by bumetanide remains to be determined.

The ethacrynic acid block of VGSC (IC_50_ = 36 μM) was more potent than its block of NKCC1 but comparable to its block of NKCC2. Like bumetanide, EA failed to completely block VGSC currents, with approximately 20% of the current remaining at 1 mM EA.

Inhibition of voltage-gated sodium channels by BMN and EA did not alter [Na^+^]_i_ accumulation caused by ischemia-acidosis. Neither the initial rapid rise in [Na^+^]_i_ due to ischemia-acidosis, nor the slow rise in [Na^+^]_i_ during the ischemic event were reduced by either compound at concentrations that significantly inhibit VGSC (100 μM). Both ischemia and acidosis are known to evoke increases in [Na^+^]_i_ resulting in reverse-mode activity of the Na^+^/Ca^2+^ exchanger (Lenart et al., 2004; Kintner et al., 2007; Luo et al., 2008). Extrusion of Na^+^ by NCX produces Ca^2+^ influx and [Ca^2+^]_i_ elevations. Given [Na^+^]_i_ is not affected by the loop diuretics, it does not appear a reduction in [Na^+^]_i_ produced by inhibition of VGSC and concomitant lessening of Ca^2+^ influx via NCX can explain the ability of these loop diuretics to mitigate ischemia-acidosis evoked [Ca^2+^]_i_ overload. NKCC activity has been implicated in the [Na^+^]_i_ accumulation that precedes NCX activity and [Ca^2+^]_i_ overload in astrocytes (Lenart et al., 2004; Kintner et al., 2007; Luo et al., 2008). Neither bumetanide nor ethacrynic acid reduced the [Na^+^]_i_ elevation in neurons suggesting NKCC is not a major conduit for ischemia-acidosis induced Na^+^ influx which leads to [Ca^2+^]_i_ overload. The lack of NKCC contribution to [Na^+^]_i_ increases may be due to reduced activity of the cotransporter during ischemia-acidosis. It was previously reported the activity of NKCC1 is reduced by ∼80% when extracellular pH approaches 6.0 (Hegde and Palfrey, 1992).

Stroke-induced gray and white matter injury in mice was shown to be reduced in NKCC knockout animals (H. Chen, Luo, Kintner, Shull, and Sun, 2005). Similarly, bumetanide was shown to reduce infarct volume in a rat middle cerebral artery occlusion stroke model (O'Donnell, Tran, Lam, Liu, and Anderson, 2004). NKCC1 effects on [Na^+^]_i_ in neurons and astrocytes appeared during re-oxygenation rather than during the ischemic event (H. Chen et al., 2005), consistent with our observation that bumetanide does not reduce [Na^+^]_i_ accumulation during ischemia-acidosis. While reduced edema associated with NKCC1 inhibition by bumetanide may lessen stroke injury (O'Donnell et al., 2004), the blunting of [Ca^2+^]_i_ overload in response to ischemia-acidosis by BMN would also improve outcomes in stroke. Similarly, inhibition of voltage-gated channels may explain how bumetanide reduces glutamate-mediated excitotoxicity (Beck, Lenart, Kintner, and Sun, 2003).

Bumetanide has been shown to decrease seizure activity in humans (Kahle and Staley, 2008). Results from the current study suggest this effect may be due to the inhibition of voltage-gated channels. The inhibition of voltage-gated Ca^2+^ channels is known to contribute to the actions of antiepileptic drugs, such as levetiracetam (Niespodziany, Klitgaard, and Margineanu, 2001; Yan et al., 2013). Similarly, inhibiting TTX-sensitive sodium channels, such as NaV1.6 has been shown to blunt seizure activity in rat epilepsy models (Hargus, Nigam, Bertram, and Patel, 2013; Shao et al., 2017). Finally, loop diuretics, including bumetanide, have been shown to promote direct vasorelaxation in the concentration range shown here to be effective for VGCC and VGSC block (Pickkers, Russel, Thien, Hughes, and Smits, 2003). Therefore, direct inhibition of these channels which contribute to vascular tone may explain these effects.

Clinically, bumetanide and ethacrynic acid, are used in edematous states, such as heart failure, to promote diuresis and natriuresis (Somberg and Molnar, 2009). This effect is due to the ability of these compounds to block the NKCC2 cotransporter, primarily in the Loop of Henle, and prevent the reuptake of Na^+^, K^+^, and Cl^−^ from the tubular fluid. Loop diuretics have additional effects, such as the lowering of blood pressure, which is often observed even prior to diuresis (Gabriel, 1983). The effective concentrations of bumetanide and ethacrynic acid reported here are consistent with clinically relevant doses (Ward and Heel, 1984; Lacreta et al., 1994; van der Heijden et al., 1998), and thus may contribute to the systemic effects of these compounds. It will be important to determine if other off-target effects of these loop diuretics contribute to the decrease in [Ca^2+^]_i_ overload reported here.

In conclusion, the loop diuretics, bumetanide and ethacrynic acid effectively suppress ischemia-acidosis induced [Ca^2+^]_i_ overload in neurons at concentrations near their respective IC_50_ values for NKCC inhibition. These effects appear to be in part mediated via the inhibition of voltage-gated Ca^2+^ and Na^+^ channels and are not due to any direct effects on ionotropic glutamatergic receptors or acid-sensing ion channels. Furthermore, the inability of the loop diuretics to reduce ischemia-acidosis evoked [Na^+^]_i_ elevations suggest NKCC cotransporters are not involved in neuronal ionic imbalances during ischemia-acidosis; and inhibition of these transporters does not account for the beneficial effects of bumetanide and ethacrynic acid under these conditions. However, the ability of these loop diuretics to lessen ischemia-acidosis induced [Ca^2+^]_i_ overload suggests that they may be useful for reducing stroke injury and that their effect on Ca^2+^ and Na^+^ channels may in part explain observations made in previous studies.

## Data Availability

The raw data supporting the conclusions of this article will be made available by the authors, without undue reservation.

## Supplementary Material

SUPPLEMENTARY FIGURE S1 Bumetanide and Ethacrynic acid inhibition of voltage-gated sodium and calcium channels does not shift the voltages of maximal current activation. **(A)**, Current-voltage relationships of mean peak whole-cell VGSC currents (mean ± SEM, *n* = 27) recorded from neurons in response to 50 msec steps from a holding potential of −70 mV to test potentials from −70 to + 60 mV in both the absence (Control, black) and presence of 56 μM ethacrynic acid (56 μM EA, red). **(B)**, Current-voltage relationships of mean peak whole-cell VGSC currents (mean ± SEM, *n* = 12) recorded from neurons in response to 50 msec steps from a holding potential of −70 mV to test potentials from −50 to +5 mV in both the absence (Control, black) and presence of 30 μM bumetanide acid (30 μM BMN, blue). **(C)**, Current-voltage relationships of mean peak whole-cell VGCC currents (mean ± SEM, *n* = 11) recorded from neurons in response to 500 msec steps from a holding potential of −70 mV to test potentials from −60 to + 40 mV in both the absence (Control, black) and presence of 10 μM ethacrynic acid (10 μM EA, red). **(D)**, Current-voltage relationships of mean peak whole-cell VGCC currents (mean ± SEM, *n* = 6) recorded from neurons in response to 500 msec steps from a holding potential of −70 mV to test potentials from −40 to +10 mV in both the absence (Control, black) and presence of 10 μM bumetanide (10 μM BMN, blue).
