# Supplementary figures and images for: Loop Diuretics Inhibit Ischemia-Induced Intracellular Ca2+ Overload in Neurons via the Inhibition of Voltage-Gated Ca2+ and Na+ Channels
Source: Front Pharmacol. 2021 Sep 15;12:732922. doi: 10.3389/fphar.2021.732922 (PMC8479115; doi:10.3389/fphar.2021.732922)

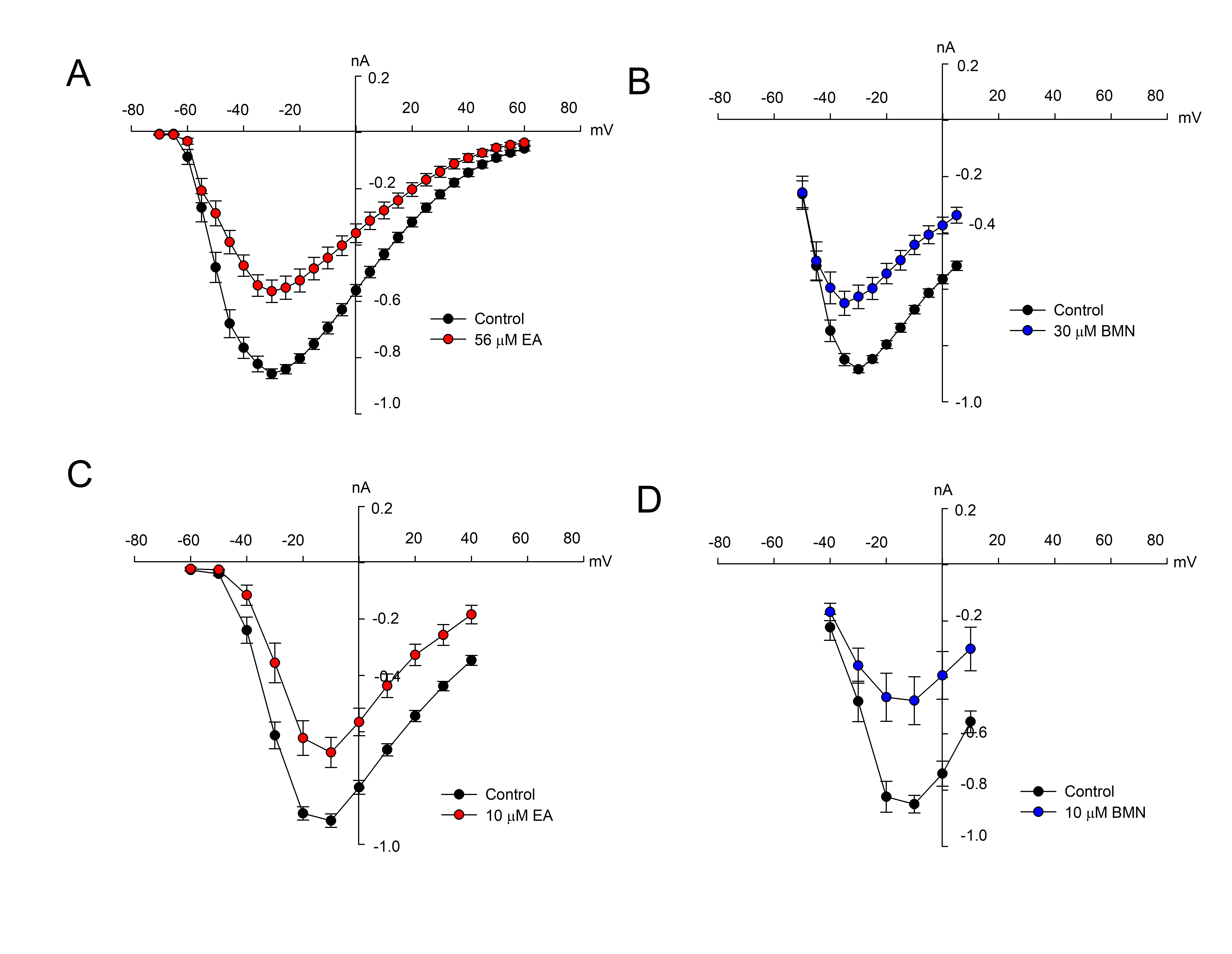

Supplement: Supplementary file 1 [file Image1.jpg]
