# Supplementary material for: Loop Diuretics Inhibit Ischemia-Induced Intracellular Ca2+ Overload in Neurons via the Inhibition of Voltage-Gated Ca2+ and Na+ Channels
Source: Front Pharmacol. 2021 Sep 15;12:732922. doi: 10.3389/fphar.2021.732922 (PMC8479115; doi:10.3389/fphar.2021.732922)
Supplement: Supplementary file 2 [file DataSheet1.docx]

Supplementary Figure 1. Bumetanide and Ethacrynic acid inhibition of voltage-gated sodium and calcium channels does not shift the voltages of maximal current activation. A, Current-voltage relationships of mean peak whole-cell VGSC currents (mean ± SEM, n=27) recorded from neurons in response to 50 msec steps from a holding potential of -70 mV to test potentials from -70 to +60 mV in both the absence (Control, black) and presence of 56 μM ethacrynic acid (56 μM EA, red). B, Current-voltage relationships of mean peak whole-cell VGSC currents (mean ± SEM, n=12) recorded from neurons in response to 50 msec steps from a holding potential of -70 mV to test potentials from -50 to +5 mV in both the absence (Control, black) and presence of 30 μM bumetanide acid (30 μM BMN, blue). C, Current-voltage relationships of mean peak whole-cell VGCC currents (mean ± SEM, n=11) recorded from neurons in response to 500 msec steps from a holding potential of -70 mV to test potentials from -60 to +40 mV in both the absence (Control, black) and presence of 10 μM ethacrynic acid (10 μM EA, red). D, Current-voltage relationships of mean peak whole-cell VGCC currents (mean ± SEM, n=6) recorded from neurons in response to 500 msec steps from a holding potential of -70 mV to test potentials from -40 to +10 mV in both the absence (Control, black) and presence of 10 μM bumetanide (10 μM BMN, blue).
